# Human T-cell Lymphotropic Virus Type 1 (HTLV-1)-Associated Adult T-cell Leukemia/Lymphoma in a Patient Previously Treated for Strongyloidiasis

**DOI:** 10.7759/cureus.47283

**Published:** 2023-10-18

**Authors:** Anam Basharat, Muhammad Yasir Anwar, Muhammad Sulh, Sindhaghatta Venkatram, Gilda Diaz-Fuentes

**Affiliations:** 1 Department of Internal Medicine, BronxCare Health System, Bronx, USA; 2 Department of Pathology, BronxCare Health System, Bronx, USA; 3 Department of Pulmonary and Critical Care Medicine, BronxCare Health System, Bronx, USA

**Keywords:** htlv-1 associated atll, strongyloidiasis, hypercalcemia in atll, rash, lymphadenopathy

## Abstract

Adult T-cell leukemia/lymphoma (ATLL) is a rare form of T-cell lymphoma with poor median survival time and limited response to chemotherapy. We present a 45-year-old female from Ghana with generalized body rash, hypercalcemia, lymphadenopathy, and lytic bone lesions. She had a history of strongyloidiasis, treated two years ago, and her serology was positive for the human T-cell lymphotropic virus type 1 (HTLV-1). Histopathology of cervical lymph node and abdominal rash biopsy revealed T-cell lymphoma. We present a literature review on the topic and the challenges of diagnosis. We emphasize the importance of considering HTLV-1-associated ATLL in patients who have been treated for strongyloidiasis in the past and are presenting with rash or lymphadenopathy.

## Introduction

Pruritic skin rash is a common presenting symptom encountered by multiple medical specialties. The differential diagnosis of a pruritic rash is broad and includes conditions such as dermatitis (atopic or contact), urticaria, psoriasis, scabies, insect bites, prurigo nodularis, lichen planus, and systemic conditions like cholestasis, renal failure, Hodgkin's lymphoma, polycythemia vera, and solid tumors [1].

The occurrence of malignancy with initial skin manifestations is uncommon. Skin manifestations of internal malignancies can arise directly from tumor invasion or metastases or indirectly through triggers that cause cutaneous signs unrelated to the primary tumor. These can occur alone or as components of paraneoplastic syndromes. In either scenario, they suggest an underlying neoplasm [2].

Adult T-cell leukemia/lymphoma (ATLL) is a rare form of lymphoma caused by the human T-cell lymphotropic virus type 1 (HTLV-1) [3]. It was first described in 1977 by Dr. Takatsuki and his colleagues in Japan. Their work was crucial in recognizing this distinct clinical entity and its association with HTLV-1 infection [4]. Approximately 1% to 5% of individuals infected with HTLV-1 are estimated to eventually develop ATLL, and usually, there is a long latency (usually 20-30 years) between infection and the development of lymphoma [5].

Our patient represents a rare form of non-Hodgkin lymphoma, ATLL, associated with HTLV-1 infection and strongyloidiasis [6].

## Case presentation

A 45-year-old female from Ghana presented to the emergency department (ED) with a worsening pruritic rash of two weeks duration associated with weight loss. She immigrated to the United States around five years back, and her medical history was relevant for hypertension, pre-diabetes, lumbar spine radiculopathy with disk herniation, Crohn's disease, tension headaches, asymptomatic thymic enlargement, and treated strongyloidiasis (2021) and Helicobacter pylori infection (2018). She abstains from smoking and alcohol and denies any drug abuse.

**Figure 1:**
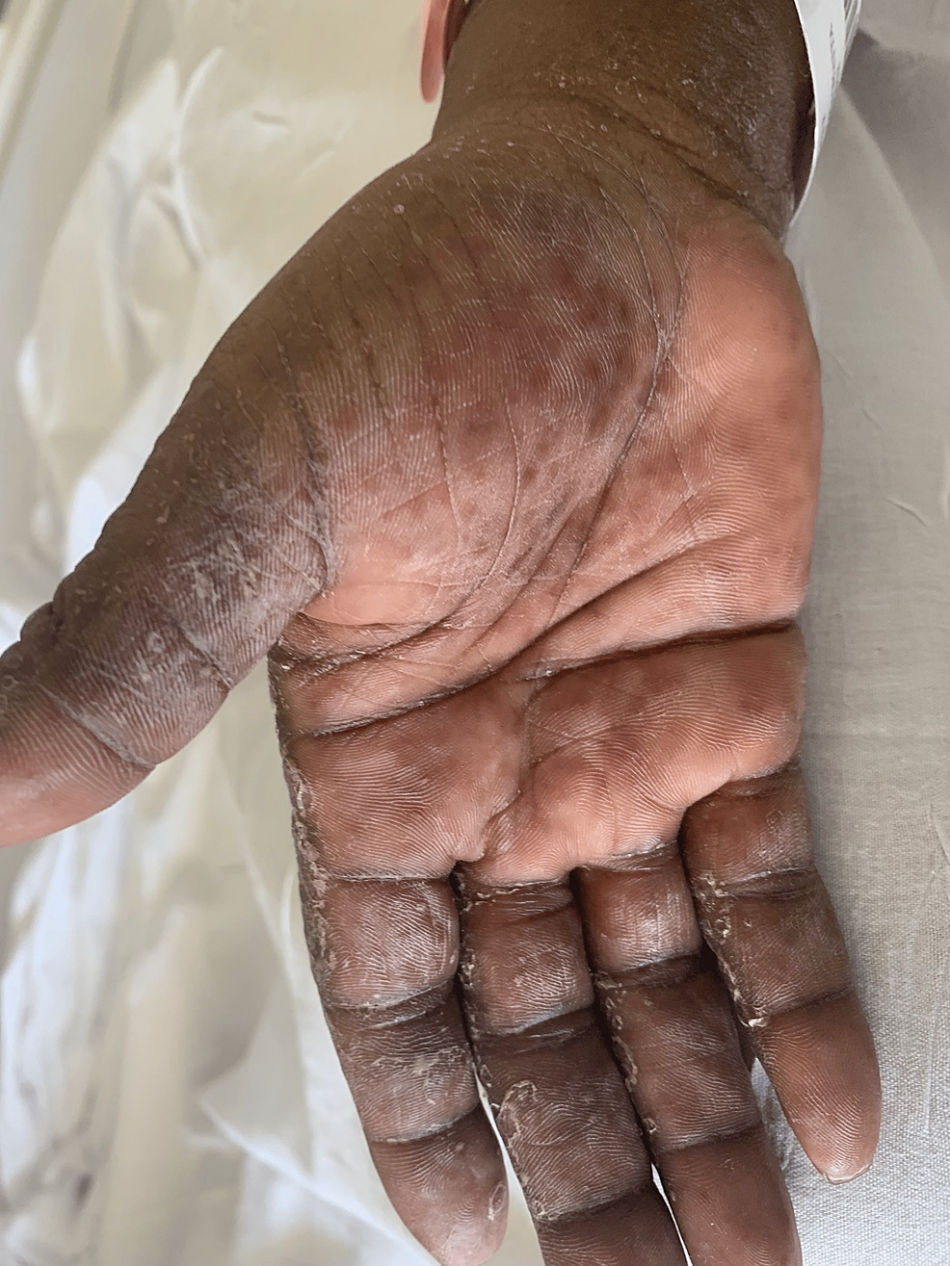
Rashes on the hand.

**Figure 2:**
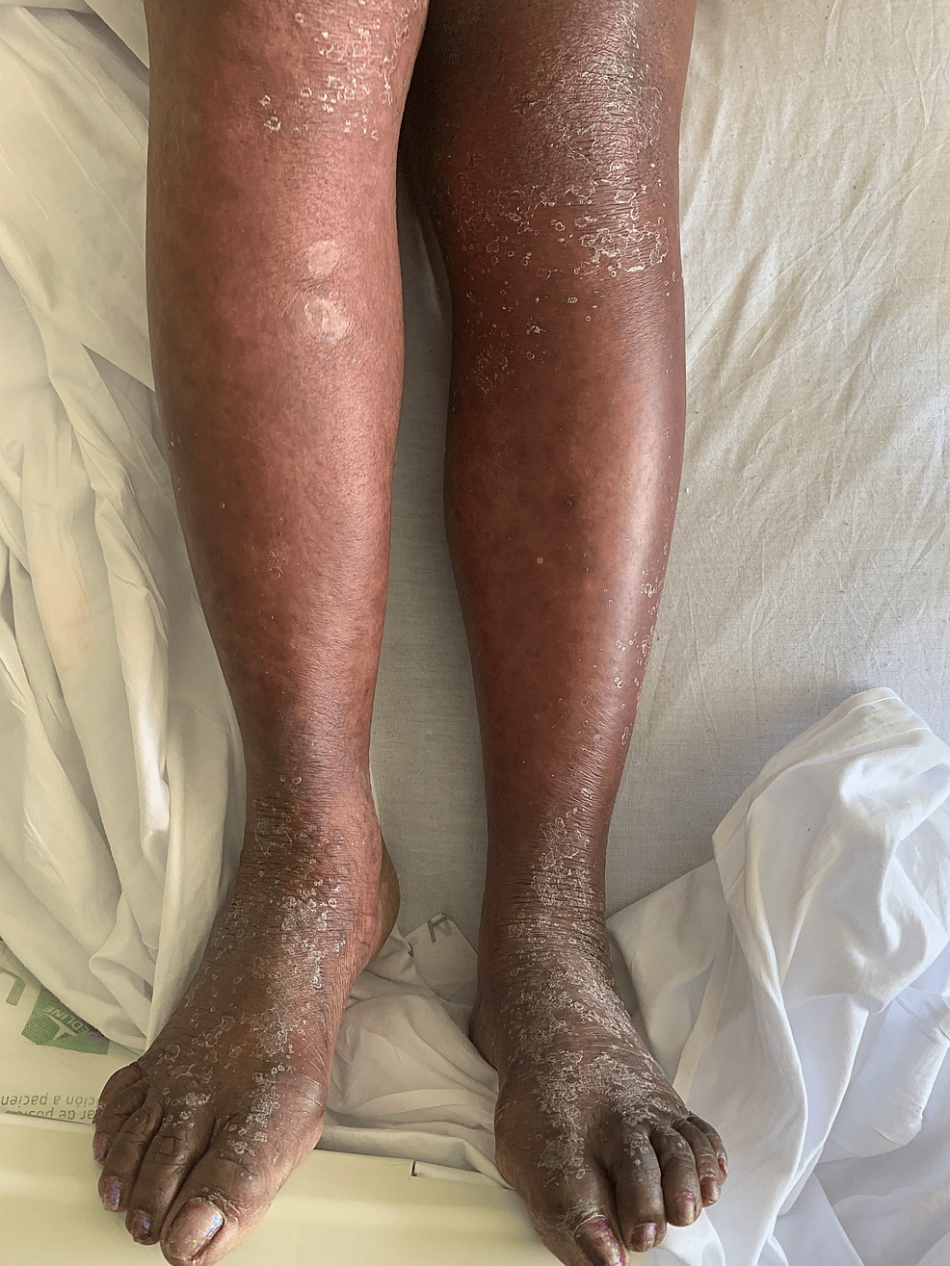
Rashes on the legs and feet.

She was started on gabapentin for neuropathy four weeks before her presentation. She reported a painful, raised, pruritic, hyperpigmented, and scaly rash on the dorsal aspect of her hands and feet. This rash later extended to involve all extremities and the abdomen, and it began soon after the initiation of gabapentin (Figures 1-2).

In the ED, she was found to be febrile (101 °F). The physical examination was remarkable for right neck swelling and widespread involvement of the body with eczematous patches with peeling.

**Figure 3:**
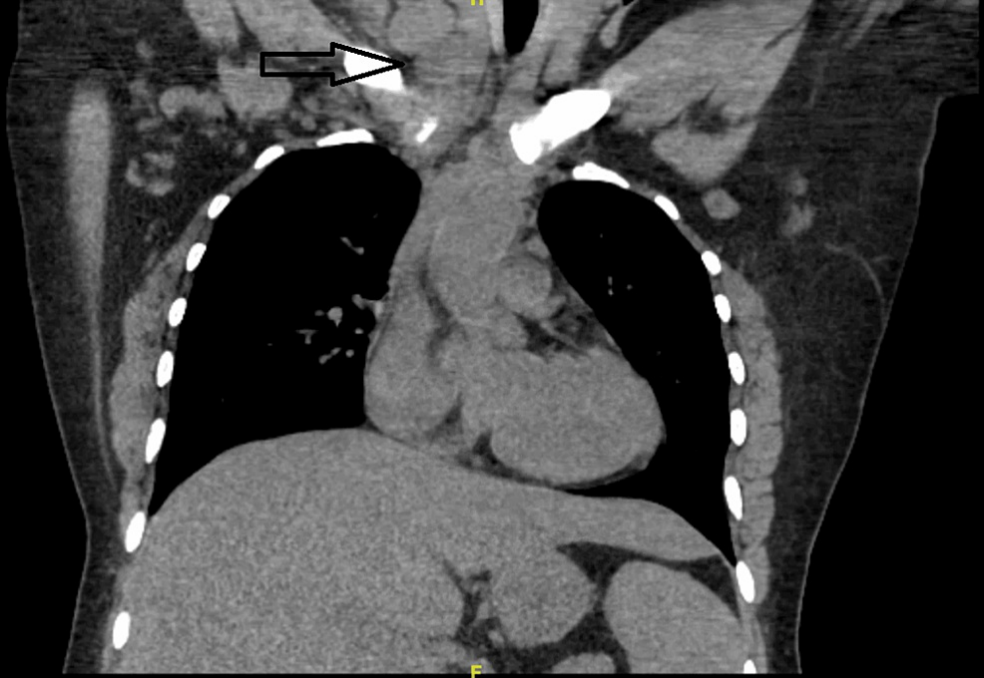
CT chest without contrast demonstrating prominent bulky lymphadenopathy in the supraclavicular regions. CT, computed tomography

The initial laboratory was significant for hypercalcemia of 14 mg/dL (normal range 8.5-10.5 mg/dL), transaminitis with alanine transaminase of 57 units/L (5-40 units/L), and aspartate aminotransferase of 59 units/L (9-36 units/L), and elevated C-reactive protein of 15.97 mg/L (<=5 mg/L). There was no serum eosinophilia. CT chest, abdomen, and pelvis showed bulky lymphadenopathy in the supraclavicular regions, multiple pulmonary nodules, bilateral iliac and groin lymphadenopathy, and innumerable lytic lesions of the bones, suggesting widespread osseous metastatic disease (Figure 3).

**Figure 4:**
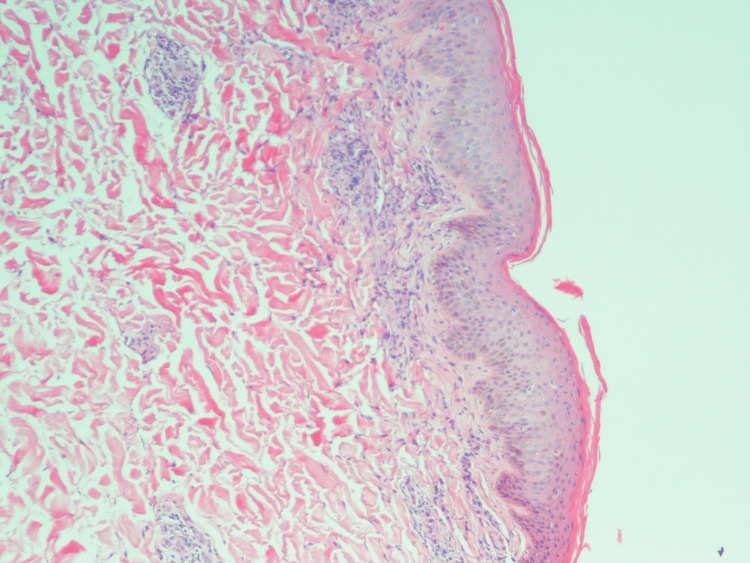
Dermal perivascular T-cell infiltrate (hematoxylin and eosin staining, magnification 10x).

**Figure 5:**
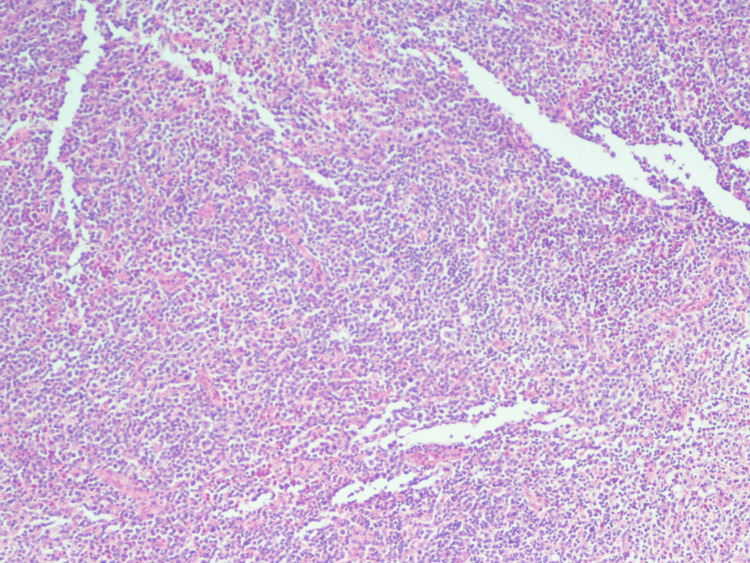
Lymph node with complete effacement of the nodal architecture by diffuse polymorphous infiltrate (hematoxylin and eosin staining, magnification 4x).

**Figure 6:**
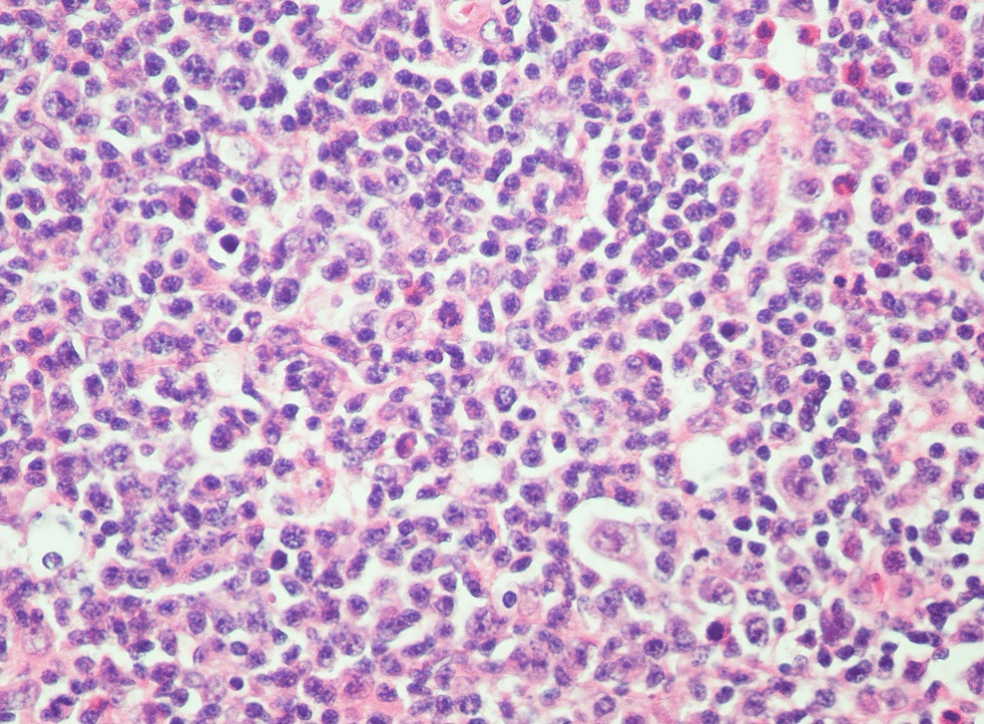
Frequent medium-to-large atypical lymphoid cells with irregular nuclei and variably prominent nucleoli (hematoxylin and eosin staining, magnification 40x).

The patient was admitted to the intensive care unit, managed for hypercalcemia with intravenous fluids, and worked up for an unexplained generalized rash. Further, the workup suggested hypercalcemia due to malignancy (parathyroid hormone was normal, and parathyroid hormone-related protein was elevated). The patient was assessed by the dermatologist, and the primary consideration was HTLV-1 lymphoma. Other differentials on the list included adult onset eczema, cutaneous T-cell lymphoma, or a potential drug eruption. She underwent a supraclavicular lymph node and abdominal skin rash biopsy, suggesting ATLL (Figures 4-6). Infectious disease specialists also evaluated her and conducted workups for sexually transmitted diseases (including HIV, syphilis, and chlamydia/gonorrhea) tuberculosis, rickettsia, and Bartonella. All septic workup was negative for bacterial, fungal, or mycobacterium infection. Her HTLV-1 was positive. A bone marrow biopsy was attempted but was unsuccessful.

The patient was stabilized and later transferred to a tertiary care hospital for chemotherapy and possible bone marrow transplantation for the ATLL. Unfortunately, the patient was later lost to follow-up.

## Discussion

ATLL is a rare form of T-cell lymphoma associated with HTLV-1 infection that can involve multiple organs. It is characterized by circulating CD4+ and CD25+ T-cells and lymphadenopathy. The incidence of ATLL varies with the demographics. The virus is usually transmitted sexually, via blood transfusion, during birth, or during breastfeeding. HTLV-1 causes a variety of diseases. The two major diseases associated with significant morbidity and mortality are ATLL or HTLV-1-associated myelopathy/tropical spastic paraparesis. Less than 5% of the people infected with the virus develop ATLL. The 2008 World Health Organization Classification of Tumors of Hematopoietic and Lymphoid Tissues subdivided ATLL into four subtypes (acute, lymphoma, chronic, and leukemia), depending upon organ involvement, hypercalcemia, LDH, and presence or absence and degree of leukemic manifestation [7]. The chronic and smoldering types are indolent but can progress to acute ATLL at times.

ATLL almost exclusively affects adults with a median age of 60 at presentation [8] but varies depending on the geographic prevalence rate of the HTLV-1 infection. There has been immunological and epidemiological overlap between strongyloidiasis and HTLV-1 infection [9]. Infection by HTLV-1 is a significant risk factor for developing Strongyloides stercoralis hyperinfection, and S. stercoralis increases the risk of developing ATLL. S. stercoralis infection stimulates the oligoclonal proliferation of HTLV-1-infected cells [10]. HTLV-1 also alters the immune response to strongyloidiasis by causing a Th2-to-Th1 shift that accounts for a high incidence of disseminated strongyloidiasis in this group of patients [11].

Intestinal strongyloidiasis as a presenting symptom of HTLV-1-associated ATLL has been described [12]. HTLV-1 co-infection with S. stercoralis has been observed in a number of published patient cases and case series [13-15]. A third of the patients with strongyloidiasis and positive serology for HTLV‐1 have a clonal integration of the provirus in their lymphocytes, leading to the hypothesis that this parasite plays a role in the development of ATLL in healthy carriers [16].

The presentation of ATLL exhibits distinct variations based on its subtypes. The acute subtype typically manifests with elevated white blood cell counts alongside potential indicators like lymphadenopathy, hepatosplenomegaly, hypercalcemia, cutaneous involvement, and in rare cases, pulmonary or central nervous system symptoms. Meanwhile, the lymphomatous subtype predominantly displays pronounced lymphadenopathy, often accompanied by heightened levels of lactate dehydrogenase (LDH) and calcium. In contrast, the chronic subtype is characterized by cutaneous involvement and lymphadenopathy. This subtype can also feature elevated white blood cell counts, low serum albumin, increased LDH, and blood urea nitrogen. Conversely, the smoldering subtype frequently remains asymptomatic, with its primary presentations centered around cutaneous and pulmonary symptoms. An additional provisional subtype, known as primary cutaneous tumoral ATLL, presents as tumors and nodules. Notably, the cutaneous manifestations in ATLL encompass a polymorphous spectrum, ranging from patches, plaques, nodules, and tumors to erythroderma and purpura [17]. This diverse range of presentations underscores the complexity and heterogeneity of ATLL's clinical landscape [2].

ATLL's differentiation from other conditions remains a complex challenge due to the lack of definitive clinical markers. Diagnosing ATLL necessitates a comprehensive assessment, considering the distinct clinical presentations, intricate morphological traits, and immunophenotypic characteristics of malignant cells, alongside the confirmation of HTLV-1 infection [18]. In acute, chronic, and smoldering subtypes, identifying a small, 5% of tumor cells through cytological and immunophenotypic analysis in the blood, combined with confirmed HTLV-1 infection, proves sufficient for diagnosis. In contrast, the lymphomatous subtype requires an excisional biopsy of the involved lymph node for a comprehensive histopathological evaluation. The presence of flower cells or clover cells is regarded as pathognomonic for ATLL.

Due to the rarity of ATLL, there have been limited trials to establish its management, so most of the therapies are the same as those for other types of T-cell lymphoma. The treatment is based on the ATLL subtype. The first-line treatment modality for acute and lymphomatous types of ATLL is chemotherapy or an antiviral regimen with close monitoring. On the other hand, watchful waiting is implemented for smoldering and chronic types.

The leukemic form of ATLL has better outcomes with antiviral therapy (zidovudine and interferon), while the lymphomatous form is more responsive to chemotherapy. Several chemotherapy regimens are available, but the VCAP-AMP-VECP combination is currently the standard regimen for aggressive ATLL in Japan. Alternative regimens in the United States include dose-adjusted EPOCH (etoposide phosphate, vincristine sulfate, cyclophosphamide, and doxorubicin hydrochloride [hydroxy-daunomycin]), CHOP (cyclophosphamide, doxorubicin hydrochloride [hydroxydaunomycin], vincristine sulfate, and prednisone), and hyper-CVAD (cyclophosphamide, vincristine, doxorubicin, and dexamethasone) [19,20].

Hypercalcemia is the most frequent complication of ATLL, and about 70% of ATLL patients develop hypercalcemia during illness [21]. The hypercalcemia is likely paraneoplastic due to the release of chemokines from malignant cells, parathyroid hormone-related peptide, tumor necrosis factor beta or interleukin-1, or increased receptor activator of nuclear factor kappa-Β ligand (RANKL) [22]. Intravenous (IV) fluids, calcitonin, IV bisphosphonate, denosumab, corticosteroids, and cinacalcet constitute the fundamental treatment approaches for managing malignancy-associated hypercalcemia. In adults with severe hypercalcemia, combining calcitonin with either an IV bisphosphonate or denosumab is recommended [23].

Prognosis depends on the subtype, with the worst prognosis for the acute and lymphoma subtypes compared with the chronic and smoldering ones. A 1984-1987 Lymphoma Study Group reported 6-, 10-, and 24-month median survival times for acute, lymphoma, and chronic types, respectively. Poor prognostic factors include advanced performance status (based on the five-grade scale of the World Health Organization), high LDH, age ≥ 40 years, ≥3 involved lesions, hypercalcemia, and others. In a 2000-2009 retrospective study including patients treated with intensive combination chemotherapy and allogeneic hematopoietic stem cell transplantation, median survival times were 8.3, 10.6, 31.5, and 55 months for acute, lymphoma, chronic, and smoldering types, respectively. The four-year overall survival rates for these subtypes were 11%, 16%, 36%, and 52% [24].

Recent research established distinct survival times for different risk levels in acute and lymphoma subtypes. Other factors include bone marrow and skin involvement, monocytosis, eosinophilia, high LDH, blood urea nitrogen (BUN), low albumin, interleukin-5, CCR4 expression, p53 mutation, p16 deletion, nuclear c-Rel, and IRF-4 expression. These factors illuminate ATLL's intricate prognosis assessment [25]. A common cause of mortality in patients with ATLL is attributed to profound immunosuppression, which increases susceptibility to opportunistic infections and related complications.

## Conclusions

Diagnosis of ATLL is challenging due to the rarity of the disease and the variety of presentations. Skin rashes and parasitic infections are usually considered benign conditions and are managed by various medical specialties.

Hematological conditions, such as in our patient with ATLL, carry a poor prognosis. Early suspicion, diagnosis, and treatment could significantly improve the overall outcome. Patients presenting with a rash should undergo a detailed examination, including an assessment for lymphadenopathy and organomegaly, along with a thorough review of past parasitic infections. Close follow-up is required to order pertinent diagnostic tests. 
